# Mechanoreceptor sensory feedback is impaired by pressure induced cutaneous ischemia on the human foot sole and can predict cutaneous microvascular reactivity

**DOI:** 10.3389/fnins.2024.1329832

**Published:** 2024-04-02

**Authors:** Erika E. Howe, Michael Apollinaro, Leah R. Bent

**Affiliations:** Department of Human Health and Nutritional Sciences, University of Guelph, Guelph, ON, Canada

**Keywords:** skin, microvasculature, post-occlusive reactive hyperaemia, foot sole, tactile perception, ulcer prevention, ischemia

## Abstract

**Introduction:**

The foot sole endures high magnitudes of pressure for sustained periods which results in transient but habitual cutaneous ischemia. Upon unloading, microvascular reactivity in cutaneous capillaries generates an influx of blood flow (PORH: post-occlusive reactive hyperemia). Whether pressure induced cutaneous ischemia from loading the foot sole impacts mechanoreceptor sensitivity remains unknown.

**Methods:**

Pressure induced ischemia was attained using a custom-built-loading device that applied load to the whole right foot sole at 2 magnitudes (15 or 50% body weight), for 2 durations (2 or 10 minutes) in thirteen seated participants. Mechanoreceptor sensitivity was assessed using Semmes-Weinstein monofilaments over the third metatarsal (3MT), medial arch (MA), and heel. Perceptual thresholds (PT) were determined for each site prior to loading and then applied repeatedly to a metronome to establish the time course to return to PT upon unload, defined as PT recovery time. Microvascular flux was recorded from an in-line laser speckle contrast imager (FLPI-2, Moor Instruments Inc.) to establish PORH peak and recovery rates at each site.

**Results:**

PT recovery and PORH recovery rate were most influenced at the heel and by load duration rather than load magnitude. PT recovery time at the heel was significantly longer with 10 minutes of loading, regardless of magnitude. Heel PORH recovery rate was significantly slower with 10minutes of loading. The 3MT PT recovery time was only longer after 10 minutes of loading at 50% body weight. Microvascular reactivity or sensitivity was not influenced with loading at the MA. A simple linear regression found that PORH recovery rate could predict PT recovery time at the heel (*R*^2^=0.184, *p*<0.001).

**Conclusion:**

In populations with degraded sensory feedback, such as diabetic neuropathy, the risk for ulcer development is heightened. Our work demonstrated that prolonged loading in healthy individuals can impair skin sensitivity, which highlights the risks of prolonged loading and is likely exacerbated in diabetes. Understanding the direct association between sensory function and microvascular reactivity in age and diabetes related nerve damage, could help detect early progressions of neuropathy and mitigate ulcer development.

## Introduction

Skin on the foot sole acts as a protective barrier and it also enables us to interact with our environment through sensory feedback. Cutaneous mechanoreceptors in the feet provide information on contact force, pressure, slips and vibration and as such, are vital to postural control, balance, and gait (Nurse and Nigg, 2001; Eils et al., 2004; Abraira and Ginty, 2013; Zehr et al., 2014). Additionally, cutaneous input also relays important protective sensations that are necessary for withdrawal or offloading behaviors to avoid or prevent foot injury (Willer et al., 1978; Spaich et al., 2004; Nagi et al., 2019). With bipedal stance and ambulation, the skin on the foot sole must withstand high magnitudes of pressure, sometimes for prolonged periods of time. In fact, capillary blood flow in the heel of the foot is physically occluded with as little as 15% of an individual’s body weight (Chang et al., 1996). The foot will experience pressure magnitudes much larger, thus hypoxia in the foot sole skin is habitual and frequent, yet healthy feet do not ulcerate. In healthy skin, the microvasculature has a protective stress response known as post-occlusive reactive hyperemia (PORH) which is characterized by a large influx of capillary blood flow (vasodilation) upon the release of an occlusion (Johnson et al., 2014; Balasubramanian et al., 2021). This reactive mechanism is imperative to counteract the ischemic deficit and flush the hypoxic tissue with oxygen and nutrients. In addition to this robust reactive response in healthy skin, intact sensation from skin receptors generates pressure distribution variability (Niemann et al., 2020). Together PORH and skin input alleviates ischemia via hyperemia and shifts in local skin pressure.

Individuals with diabetic peripheral neuropathy have impaired sensory feedback characterized by tingling, numbness, and sometimes pain that primarily develops in the most distal aspects of the hands and feet (Zakin et al., 2019). This decrease in skin input and lack of protective sensation contributes to prolonged and excessive tissue loading (Armstrong et al., 1998, 2017; Frykberg et al., 1998; Caselli et al., 2002; Niemann et al., 2020) and is thought to be a central cause for ulcer development (Crawford et al., 2007; Armstrong et al., 2017; Zakin et al., 2019; Armstrong et al., 2023). Diabetic foot ulcers occur in as many as 25% of individuals with Diabetes (Zamzam et al., 2023) and are the leading cause for non-traumatic foot amputations in Canada (Imam et al., 2017). The pathogenesis of diabetic neuropathy is not fully understood, however, there is some evidence that the sensory end organs (mechanoreceptor) are damaged prior to any changes to axonal transduction (Mizobuchi et al., 2002). In addition to decreased sensation, individuals with diabetes also tend to have poor peripheral circulation and oxygenation of skin tissue (Jorneskog et al., 1995; Zimny et al., 2001; Petrofsky, 2012; Jan et al., 2013), which impairs skin healing (Shapiro and Nouvong, 2011; Balasubramanian et al., 2021) and further contributes to ulceration risk (Singh et al., 2005; Lim et al., 2017). PORH responses also are blunted in diabetic individuals (Petrofsky et al., 2009; Barwick et al., 2016; Lanting et al., 2017), decreasing protective stress responses against tissue loading. Interestingly, this microvascular dysfunction is already evident in individuals with obesity and prediabetes preceding any large vessel dysfunction or hyperglycemia (De Jongh et al., 2004; Stirban, 2014; Park et al., 2016). Whether the decline in function of the peripheral cutaneous sensory afferents occurs independently or consequently of the peripheral microvascular changes remains mostly conjectured. These early changes occurring in the microvasculature, local to the foot surface, make the foot skin in diabetes susceptible to persistent tissue ischemia and thus an unprecedented mix to generate sensory end-organ damage. However, investigating this time-course within a diabetic population remains problematic since the changes to vasculature or sensory end-organs have likely already begun.

Previous literature has examined the effects of a short-term ischemic cuff on cutaneous sensitivity in a healthy foot sole. Schlee and colleagues applied a pressure cuff on the thigh and examined vibration sensitivity thresholds at the heel, first metatarsal and hallux (Schlee et al., 2009). Four continuous minutes of cuff occlusion at 150 mmHg on the thigh resulted in significant decreases in perceptual sensitivity of a 200 Hz vibration across the foot sole. This finding suggests that even with healthy vasculature function, that adequate blood flow to skin tissue is imperative to perceive sensation from cutaneous sensory afferents. However, an ischemic cuff occludes arterial blood flow of the entire limb and nerve bundle, which is a more radical decrease in blood flow to more closely model large vessel vascular disease. Assessing the impact of cutaneous microvascular ischemia on cutaneous mechanoreceptors will provide an important link to the underlying progression and development of diabetic neuropathy.

The purpose of the current work is to (1) investigate the effects of localized ischemia of different magnitudes and durations to the changes in skin sensitivity and PORH responses across different sites of a healthy foot sole and (2) investigate whether changes to skin sensitivity is related to characteristics of blood flow PORH responses. It was hypothesized that longer and larger ischemic loads would generate longer durations of insensate skin, and this would strongly correlate to greater capillary PORH responses. It was also hypothesized that skin sensitivity in sites that experience higher plantar pressures during stance would be more impacted than sites than do not experience high plantar pressures. The consequences of diabetes related to foot health is both multifaceted and complex. Understanding individual contributing factors of ulceration risk, particularly those that have an early onset could be imperative for therapeutic interventions that could prevent ulcers and lower limb amputations.

## Materials and methods

### Participants

Thirteen (6 males, 7 females) healthy young adults (26.5 ± 4.2 years; BMI 24.5 ± 3.5) volunteered to partake within this study. For our study to have 80% power to detect a medium effect size (6%) with an alpha of 0.05, 13 participants were required to partake in the study. The final sample satisfied these requirements. All individuals were free from any self-reported neuromuscular, vascular or metabolic disorders. There were no reported lower limb injuries, use of any medications beyond oral contraceptives, or a history of smoking. Individuals did not perform any intense exercise or consume caffeine in the 12 h prior to testing sessions. All participants provided informed consent to participate, and procedures were approved by the University of Guelph’s Research Ethics Board.

### Experimental set up and equipment

**Figure 1:**
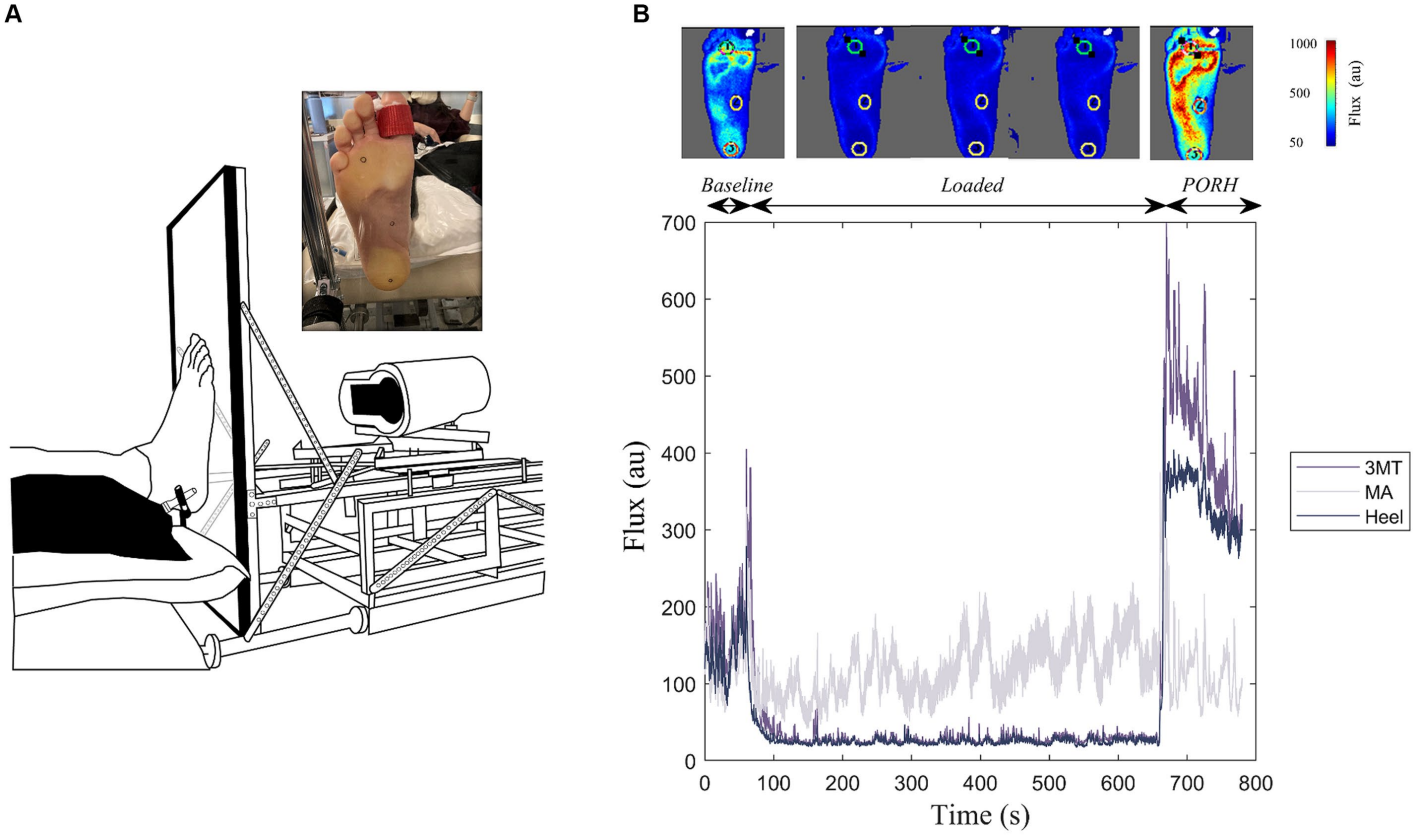
Experimental Setup. **(A)** Custom-built loading device coupled with a laser speckle contrast imager (FLPI-2, Moor Instruments Inc.) to apply standing-like load to the bottom of the right foot sole against a clear acrylic platform. Inset illustrates the foot sole marked with the regions of interest (heel, medial arch, and third metatarsal (3MT)) during the sustained load. **(B)** Example flux data for a 10-minute 50% body weight loading trial. This includes a one-minute baseline, ten-minutes of sustained loading and a two-minute PORH response for the three regions of interest. Top panel includes flux images of the whole foot at representative frames of the trial. Cooler colours indicate less flux, warmer colours are more flux.

For the laboratory experiment, participants were seated in a modular chair with their right leg extended and acclimatized for 10 min with the lights dimmed. The leg position was secured using a Versa Form pillow and a Velcro strap to avoid knee flexion during the foot loading. A custom-built motorized foot plate was fastened to the chair and was equipped with a linear actuator (IP66 PA-04, Progressive Automations) to apply a linear load to the foot (Figure 1A). An in-line force transducer (TAS501, HT-Sensor Technology Co Ltd.) was aligned in series to record the pre-determined load forces, calculated as a % body weight (BW). Force data were sampled at 12.5 Hz. The close-looped system was controlled using a microcontroller (Arduino Uno) and customized program built in the Arduino software (IDE v 1.8.19) to modulate loading speed, magnitudes, and durations.

Blood flux measures on the foot sole were measured using a laser speckle contrast imager (LSCI; MoorFLPI-2, Moor Instruments) that was also fastened to the foot plate (Figure 1A). The foot contacted a perpendicular 1.7 cm thick clear acrylic plate and remained coupled to the plate during each recording. The LSCI laser was fixed at a distance of 38 cm from the foot, as recommended by manufacturer’s instruction manual and set to a resolution of 152 × 113 pixels/cm^2^. The flux data were sampled at 25 frames/s and a time constant of 0.3 s to account for rapid blood flow changes and achieve optimum contrast through reducing the image noise (Kazmi et al., 2014). During each loading trial the foot was held in a dorsiflexed position using a strap around the big toe to prevent a foot drop with the retraction of the foot plate.

Three sites on the foot sole were examined in this study. Two of the sites, the heel and third metatarsal head (3MT), experience higher plantar pressures in standing and gait, while the medial arch (MA) is typically a lower plantar pressure site (Goffar et al., 2013; Helili et al., 2021; Cleland et al., 2023). We chose to examine the 3MT as it is a common region for foot ulcerations in pathologies such as diabetic neuropathy (Caselli et al., 2002; Petersen et al., 2020) and experiences the greatest peak pressure during gait (Melai et al., 2011; Kim et al., 2019) which is a direct predictor of ulcer formation (Pu et al., 2018).

The heel, the MA and the 3MT of the right foot sole were marked with 15 mm diameter circles using a metallic sharpie to allow these regions of interest (ROI) to be tracked on the Moor Imaging software (Figure 1A). The center of these ROI locations was measured with respect to the anatomical landmarks and borders on the foot. The heel ROI was centered around the medial-lateral and anterior–posterior borders of the heel pad. The MA ROI was marked midway of the foot length in the anterior–posterior direction and aligned with the 2nd metatarsal head in the medial-lateral axis. Lastly, the 3MT ROI was marked over the third metatarsal head. Semmes-Weinstein monofilaments were used to determine perceptual thresholds (PT) at each site. Brachial blood pressure (BPTru, BPM-200, Medical Devices, Coquitlam, Canada) was measured at the beginning and end of testing sessions to ensure central sympathetic tone was not changing over time.

### Experimental protocol

The experimental testing was completed over two separate sessions that were within 1 week of each other and began at the same time of day. The blood flux measurements from loading always occurred on test day 1 and the monofilament sensitivity measurements from loading occurred on day 2.

#### Loading protocol

The loading protocol was the same between the two test sessions and consisted of three blocks of four loading conditions. The foot was either loaded for 2 min or 10 min continuously (duration), at either 15% or 50% BW (magnitude). These four conditions were randomized within a block where all four conditions were completed before moving onto the next block. There were three blocks for a total of 12 trials. Between each block, participants were asked to stand and walk around for 2–5 min before resuming testing. At the beginning and end of each testing block, the skin temperature over each of the three sites was recorded using an infrared thermometer (CZ-IF, Thermoworks). For each trial, the motor actuator would extend until 1–2% of the participant’s BW was loaded onto the plate. This small loading magnitude was necessary to ensure that the foot remained coupled to the plate for the entire duration of the trial. The actuator would then load the foot to the appropriate magnitude (15 or 50% BW) and would sustain this for either 2 or 10 min. On the blood flux testing day, the plate would retract back to the 1–2% of BW and remain there for 2-min while the flux measurements were recorded. On the monofilament testing day, the plate would retract completely to allow access to the foot skin and measure sensitivity changes.

#### Blood flux testing

For each loading trial, once the foot was loaded at ~1–2% of the participant’s BW and the flux measurement stabilized, a 1-min baseline flux was recorded prior each load condition (2 min15%, 2 min50%, 10 min15%, 10 min50%). The plate would then retract back to the baseline load for 2 min while the PORH response was recorded from the blood flow laser speckle imager.

#### Monofilament testing

Prior to each loading trial on the monofilament testing day, PT was determined for each of the testing sites using a 4-2-1 staircase method (Dyck et al., 1993). Participant’s eyes were closed and would verbally indicate with a “Yes/No” response after a monofilament was applied perpendicular to the skin until the nylon fiber buckled. The participant’s response (yes/no) would dictate whether the subsequent monofilament applied had a smaller or larger gram force. If the monofilament could be perceived, the next monofilament applied would be smaller. The magnitude of the change in monofilament force would subsequently be reduced (4 steps, 2 steps, 1step) with each switch from perception to non-perception until PT was determined. PT was defined as the smallest gram force that the participant could confidently feel at least 75% of the time. This same monofilament identified as PT was then used to examine the time course of the skin sensitivity recovery after a local ischemia (PT recovery).

To determine PT recovery, the established PT monofilament for a site was applied in a repetitive manner to a metronome (18 bpm) for a minimum of 2 min after the load was released from the foot. The monofilament was applied after the auditory cue from the metronome beat, the participant would respond, and the beat would repeat. Individuals would respond verbally after each beat with a “Yes/No” response depending on whether they perceived the PT monofilament. If the participant did not consistently perceive the PT monofilament at the 2-min time point, the test continued until they had five consecutive affirmed perceptions.

Due to evidence of perceptual threshold drift across an experiment (Plater et al., 2021), PT for the testing site was re-established prior to each trial. In addition, it is likely the sensitivity changes due to ischemia are transient; therefore, only one site’s sensitivity recovery was tested per loading trial. The site testing order was pseudo-randomized such that each site’s change to sensitivity was tested after all the different loading conditions (see Table 1 for an example).

**Table 1:** Example for testing site order for PT recovery time.

	Loading condition			
	2 min 15%	2 min 50%	10 min 15%	10 min 50%
Block 1	3MT	Heel	MA	3MT
Block 2	MA	3MT	Heel	Heel
Block 3	Heel	MA	3MT	MA

Each site was tested once per loading condition over the three blocks. Site testing order was randomized for each participant. 3MT (third metatarsal) MA (medial arch).

### Data analysis

Flux data were processed in the moorFLPI-2 Review (V6.0) software. For each trial, flux values for the three sites were evaluated during the PORH response once the load was released (Figure 1B). PeakPORH was defined as the difference between the maximum flux value upon load release and the 1-min average loaded flux just prior. PORH recovery rate was the slope of the flux values (flux/s) over the 2-min recording. The influence of load durations and magnitudes were compared at each testing site using a 3-way MANOVA (Site*Duration* Magnitude). Follow up univariate analyses for both peakPORH and PORH recovery rate were performed with a Tukey adjustment.

Force data on each testing day was averaged to determine the mean baseline force, the loaded force and the PORH force. Force values were not statistically compared but were examined to determine that adequate force magnitudes were reached for each trial. If forces were more than 5% BW below or above the target force, data from that trial was removed from any analyses.

PT drift was assessed using a two-way ANOVA (Site*Block) to determine whether the sensitivity threshold was significantly changing over time. For PT recovery, monofilament perception responses were evaluated by first calculating a moving average of monofilament perception responses. Values of 1 indicated a percept, whereas a value of 0 was designated a non-percept. The moving average was calculated over 5 applications. This PT probability was used to establish PT recovery time. PT recovery time was defined as a PT probability of >60% which would indicate 3 or more perceptions in any given 5 consecutive trials. The first instance in time that the PT probability was >60% was defined as the PT recovery time. A 3-way repeated measures ANOVA (Site*Duration*Magnitude) assessed the effect of site and loading characteristics on monofilament sensitivity changes.

Lastly, simple linear regressions at each skin site examined the relationship and potential ability of the PT recovery time to predict the PORH flux response characteristics. All data were analyzed in a custom MATLAB program (MATLAB 2021b). Statistical testing was completed using R Studio. Significance was set at an alpha of 0.05.

## Results

All data were collected in a laboratory space with an ambient temperature of 23.3 ± 0.97°C, that remained consistent between all testing conditions and participants (*p* = 0.868). The average skin temperature on the heel, MA and 3MT decreased an average of 3.0 ± 1.9°C, 2.4 ± 1.9°C, and 2.1 ± 1.8°C, respectively, from the beginning to the end of the experiment. However, there were no significant main effects of site (*F*_2,72_ = 1.76, *p* = 0.18), test day (*F*_1,72_ = 2.02, *p* = 0.16) or interaction effect (*F*_2,72_ = 0.78, *p* = 0.73) for temperature. Blood pressure readings averaged at 115/75 mm Hg at the beginning and 116/75 mm Hg at the end of the blood flux testing session. Paired t-tests did not reveal any significant changes of systolic blood pressure (*p* = 0.19) or diastolic blood pressure (*p* = 0.35) over time. Target force was consistently reached for all 15% BW loading trials and was on average 14.9 ± 0.1% BW. However, for the 50% loading trials, the force applied was slightly more variable and on average was 48.2 ± 2.7% BW. In ~3% (9/312) of total number of loading trials over both testing days, data were excluded from the analysis because the force did not reach at least 45% of the individual’s BW.

### Monofilament sensitivity threshold

Monofilament PT was established for a single site immediately before the foot was loaded, to establish a baseline. Within a testing session, the heel baseline PT drifted 0.4 ± 0.75 g, whereas the MA and 3MT only drifted 0.08 ± 0.1 g and 0.13 ± 0.13 g, respectively. A two-way repeated measures ANOVA for the PT (site*block) revealed a main effect of site with a large effect size (*F*_2,13.36_ = 13.35, *p* = 0.002, *ƞ*^2^ = 0.322) but no effect of block (*F*_3,136_ = 0.458, *p* = 0.582, *ƞ*^2^ = 0.003) or any site*block interaction (*F*_6,23.64_ = 2.87, *p* = 0.069, *ƞ*^2^ = 0.02). Tukey *post hoc* comparisons for site revealed that the heel PT (0.85 ± 0.51 g) was significantly greater than both MA (0.37 ± 0.16 g, *p* = 0.003) and 3MT (0.39 ± 0.19 g, *p* = 0.003) but the MA and 3MT were not significantly different from each other (*p* = 0.99).

### Local ischemia influences monofilament PT recovery

**Figure 2:**
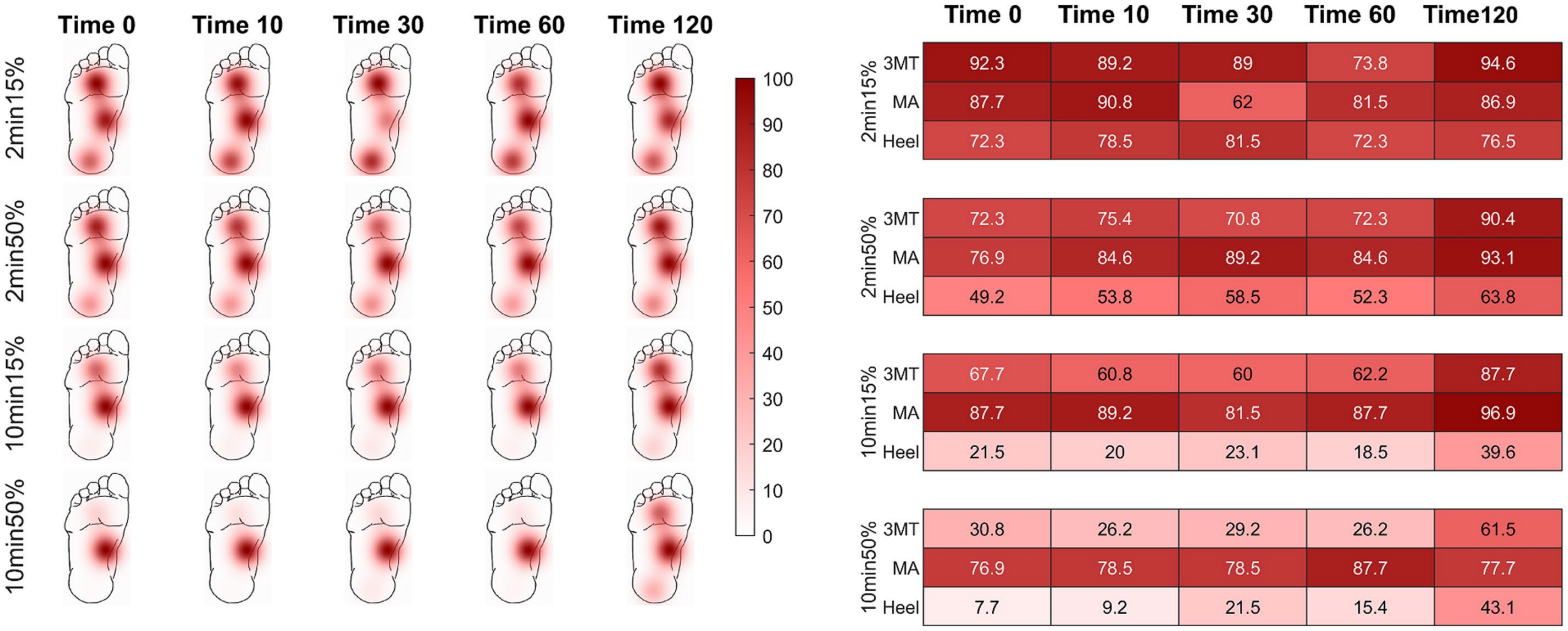
Percent of monofilament threshold perception over time using a 5-response moving average. Time of assessment is represented at 0, 10, 30, 60, and 120 seconds post load release. Recovery threshold was defined as a perception of > 60%. Darker colours indicate greater likelihood of perception. The chart provides probability values (in percentages) of sensation across all subjects for the select time points at each load condition. Initially, the two higher plantar pressure skin sites (heel and 3MT) had more unreliable perception of the monofilament than the MA, but sensation recovered as time progressed (left to right in figure). The MA had effective sensation immediately upon load release (time 0 s) for all conditions. As duration or magnitude of loading increased (top to bottom), perception of threshold was more greatly impacted.

PT probabilities across the foot sole are plotted on a time-series heat map (Figure 2). Individual heat maps represent a loading condition at select time periods after load release (0 s, 10s, 30s, 60s, and 120 s) to depict the aggregate subject averages of PT probability over time across the foot sole after the release from an ischemic load. Darker colour indicates a stronger likelihood of perception (i.e.: returned pre-load sensation) of the PT monofilament. Values of less than 60% indicate an unreliable ability to perceive the pre-loaded PT baseline. At the high pressure skin sites (3MT and heel), the PT monofilament was perceived less often upon the load release, as indicated by the smaller probability values (lighter colours) at time 0, compared to the MA sensitivity, which was consistently perceived more than 75% at time 0. When comparing across different loading durations and magnitudes, the percent of perception cumulatively decreases as the foot is loaded for 2 min to 10 min but also from 15 to 50% BW. For example, PT probability at the heel at time 0 was 72.3% for the 2 min15% loading condition but was only 49.2% with 2 min50% loading. As the duration of the loading increased to 10 min long, the monofilament was perceived at the heel 21.5% of the time with 15% BW but only 7.7% of the time after loading with 50% BW.

**Figure 3:**
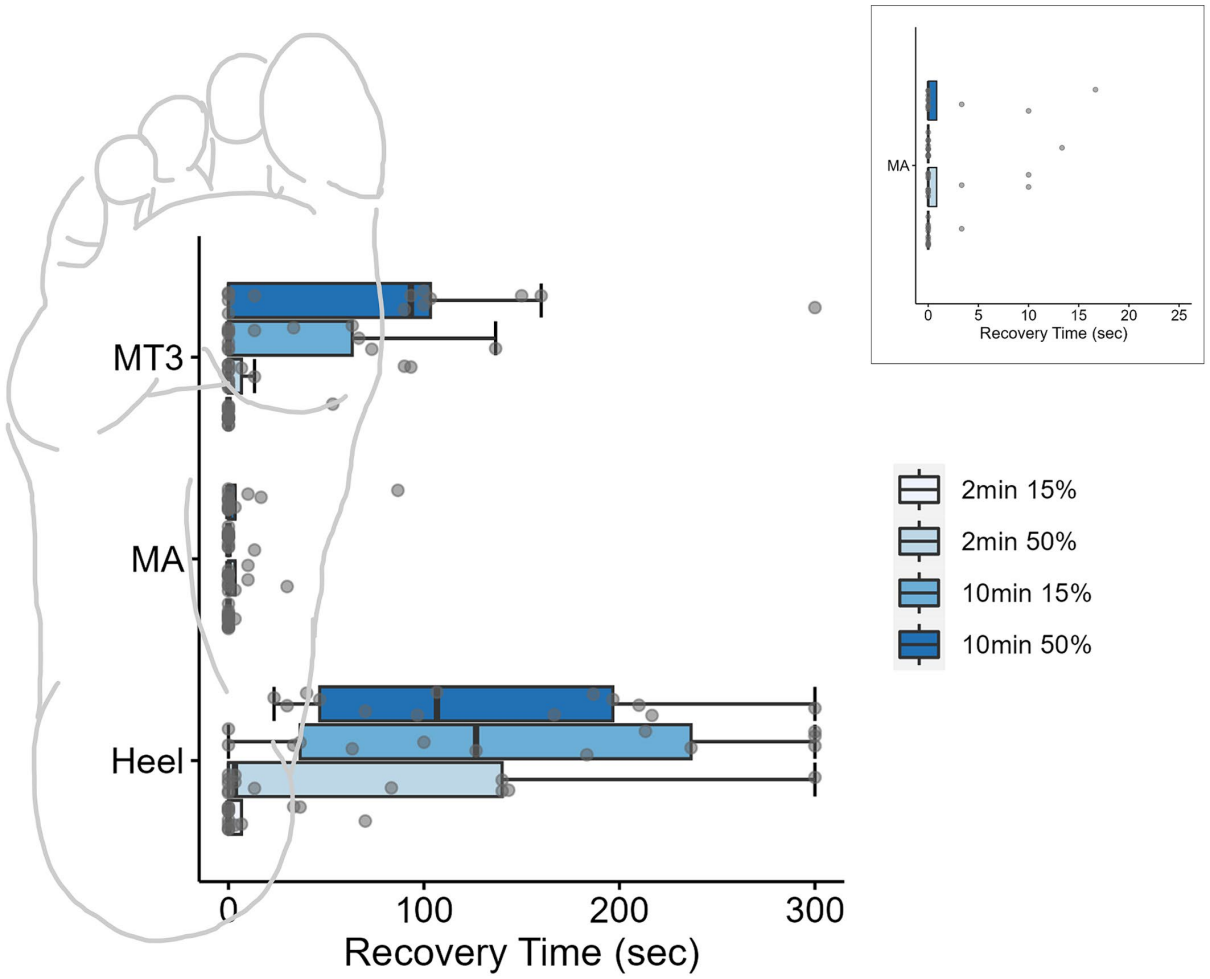
Boxplots for monofilament recovery times (sec) at each site across the foot sole after different loading conditions. Individual data points for each condition are represented by dots. Inset is the MA site magnified to display the individual data. Ten minutes of loading significantly impacted perceived sensation of the threshold monofilament at the high-pressure skin sites (heel and 3MT). Box plots are median and interquartile ranges, whiskers are the data ranges.

A three-way repeated measures ANOVA was used to evaluate the PT recovery time at each site (x3), for loading durations (x2) and loading magnitudes (x2) and revealed a significant three-way interaction of site, duration, and magnitude (*F*_2,24_ = 5.58, *p* = 0.01, *ƞ*^2^ = 0.04; Figure 3). Based on *a priori* hypotheses, only within site differences of loading conditions and between site differences within each of the four loading conditions were examined. The findings from the *post hoc* analysis using a Tukey test are outlined below.

#### Duration, but not magnitude influenced the PT recovery time

At the MA, the monofilament recovery time did not significantly vary between any of the loading magnitudes or durations (*p* > 0.05). The median PT recovery time was 0 s in all conditions, indicating that individuals would consistently perceive the PT monofilament immediately upon the release of the load (Table 2). At the 3MT, the only significant pairwise comparison of PT recovery time was observed between 2 min15% and 10 min50% loading conditions (*p* = 0.029), with a median increase in recovery of 93.3 s (Table 2; Figure 3). At the heel, PT recovery time had a median of 0 s sec with 2 min15% loading (0 ± 6.66 s) and was significantly longer after both a 10 min15% load (126.7 ± 199.9 s, *p* < 0.001) and a 10 min50% loading trial (106.7 ± 149.9 s, *p* < 0.001) but not the 2 min50% loading trial (3.3 ± 139.9 s, *p* = 0.52). The 10 min15% load was not significantly different from the 10 min50% load (*p* = 1.0).

**Table 2:** PT recovery times (sec) after sustained loading for different durations (min) and magnitudes (% body weight).

	Loading condition			
Site	2 min 15%	2 min 50%	10 min 15%	10 min 50%
Heel	0 (6.666)	3.333 (139.986)^ab^	126.654 (199.98)^abc^	106.656 (149.985)^ac^
MA	0 (0)	0 (3.333)	0 (0)	0 (3.333)
3MT	0 (0)	0 (6.666)	0 (63.327)	93.324 (103.323) ^ac^

Median (IQR) values reported. MA, medial arch. 3MT third metatarsal. ^a^Denotes significant difference from MA within a condition. ^b^Denotes significant difference from 3MT within a condition. ^c^Denotes significant difference from 2 min 15% condition within a site.

#### Third metatarsal and heel recovery time were significantly longer than the medial arch with longer loading durations

Within a single loading duration and magnitude, no differences in time to recovery were observed between sites at the 2 min15% (*p* = 0.99) or at 2 min50% (*p* = 0.65; Table 2). However, within the 10 min 15% loading condition, the heel had a significantly longer recovery time than the 3MT (*p* < 0.001) and the MA (*p* < 0.001) but the 3MT and the MA were not different from one another (*p* = 0.98). Moreover, within 10 min 50% loading condition, both the heel and the 3MT had significantly longer recovery times than the MA (*p* ≤ 0.001; *p* = 0.054 respectively). The heel and the 3MT were not different from one another (*p* = 0.74).

### PORH flux responses to a localized ischemia and its relationship with sensitivity

#### PORH peak and recovery rate are influenced by different loading characteristics

**Figure 4:**
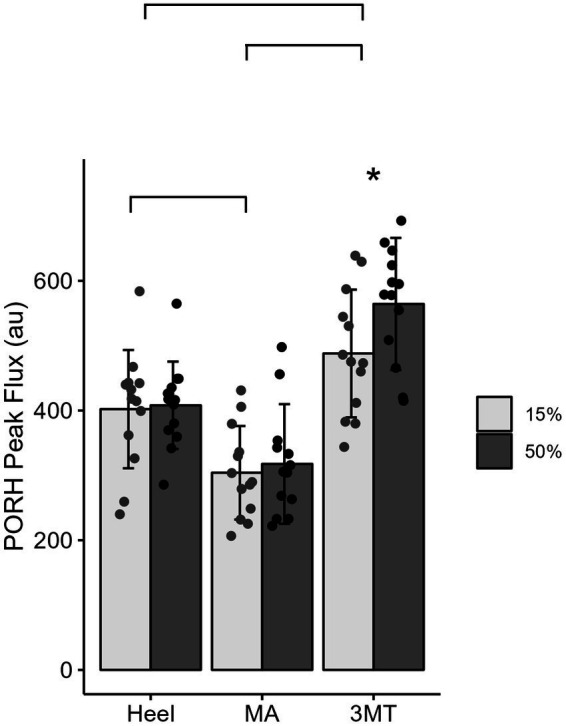
Mean ± SD of PORH Peak for each site across the foot sole for a 15% body weight load (light gray) or a 50% body weight (dark gray) load. Lines represent significant site differences. Askerisks (*) denotes a significant pairwise comparison between the 50% and 15% body weight PORH peak within the 3MT site, such that peak PORH was significantly greater with 50% body weight of loading.

**Figure 5:**
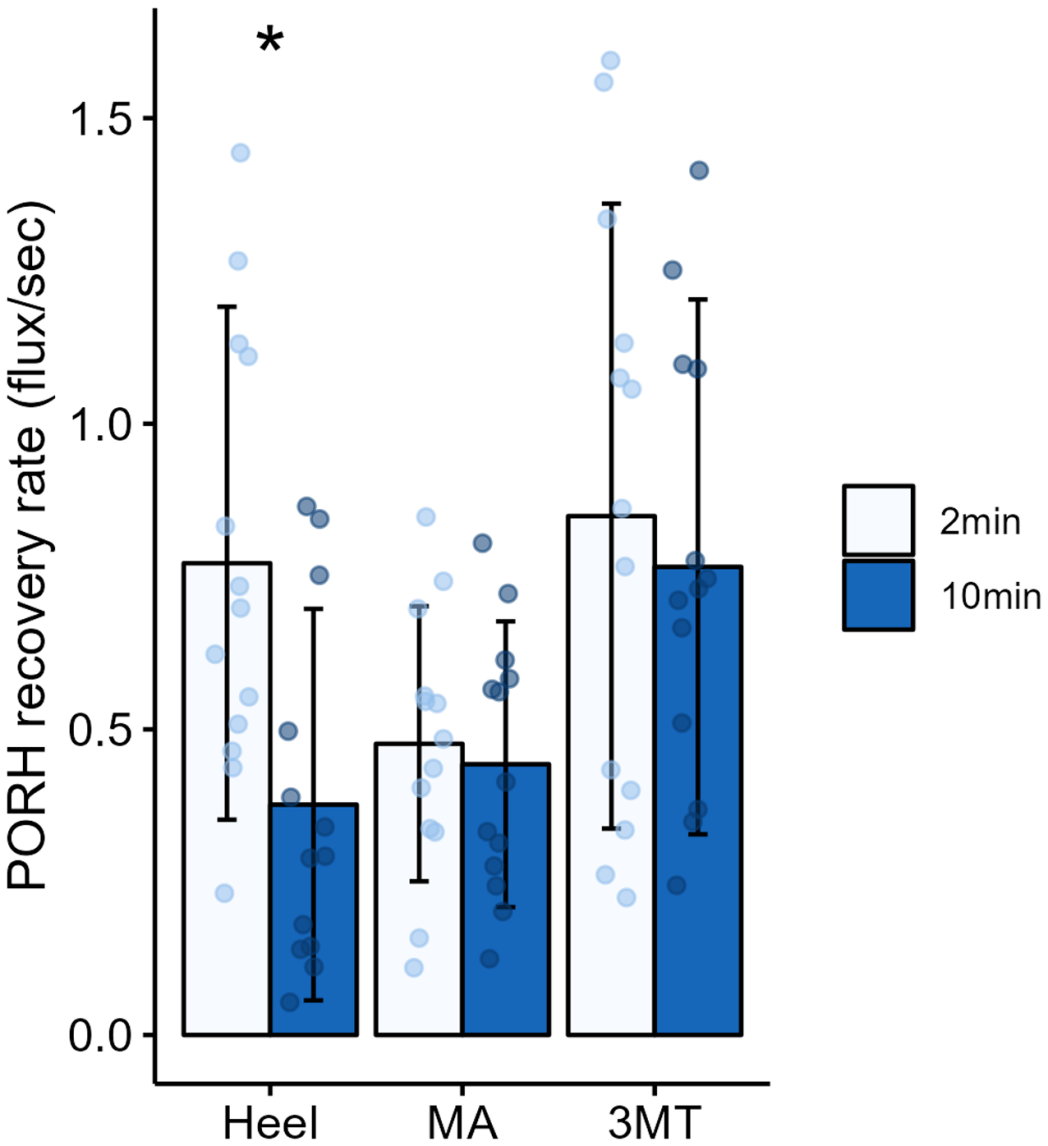
Mean ± SD of PORH recovery rates for each site across the foot sole after 2 minutes (light blue) or 10 minutes (dark blue) of sustained loading. Asterisks (*) denotes a significant pairwise comparison between 2 and 10 minutes of sustained loading, such that recovery rate was significantly slower after 10 minutes of loading on the heel.

A 3-way MANOVA for site* magnitude*duration revealed main effects for the independent factors of site (Pillai trace’s = 0.517, *F*_2, 288_ = 25.09, *p* < 0.001, *ƞ*^2^ = 0.26) and duration (Pillai trace’s = 0.079, *F*_1,143_ = 6.12, *p* = 0.003, *ƞ*^2^ = 0.08). Magnitude of loading was trending towards significance (Pillai trace’s = 0.04, *F*_1,143_ = 3.01, *p* = 0.053, *ƞ*^2^ = 0.0) and no interactions were found. Follow up univariate testing were used to determine comparisons for individual dependent variables. PeakPORH has often been used as a measure to quantify the vasodilatory capacity in skin capillaries and is primarily mediated by sensory nerve function (Minson et al., 2001, 2002; Lorenzo and Minson, 2007; Tew et al., 2011; Hodges et al., 2015; Hodges and Cheung, 2018). For peakPORH there was a main effect of both magnitude (*F*_1,144_ = 4.99, *p* = 0.027) and site (*F*_2,144_ = 76.14, *p* < 0.001). *Post-hoc* analyses revealed that peakPORH at the 3MT was significantly greater than both the MA (*p* < 0.001) and the heel (*p* < 0.001) and the peakPORH at the heel was significantly greater than at the MA (*p* < 0.001). Even though there was a significant main effect of magnitude, it was only significantly different within the 3MT, such that the 50%BW load produced a significantly larger peak than the 15% BW load (*p* = 0.029; Figure 4). Although peakPORH can indicate some extent of reactive response to a load, it fails to capture any dimension of time or duration of the reactive hyperemia. As a result, the authors wanted to investigate the PORH recovery rate to examine the rate that blood flow returns towards baseline. In contrast to the peakPORH, PORH recovery rate was not influenced by the magnitude (*F*_1,144_ = 0.005, *p* = 0.941), but rather had an interaction effect of duration*site (*F*_1,144_ = 3.52, *p* = 0.032). Pairwise comparisons found that at the heel, blood flux recovered and returned to baseline measures significantly faster with 2 min of loading compared to 10 min (*p* = 0.001; Figure 5).

#### PORH recovery rate can be predicted by monofilament PT recovery time

**Figure 6:**
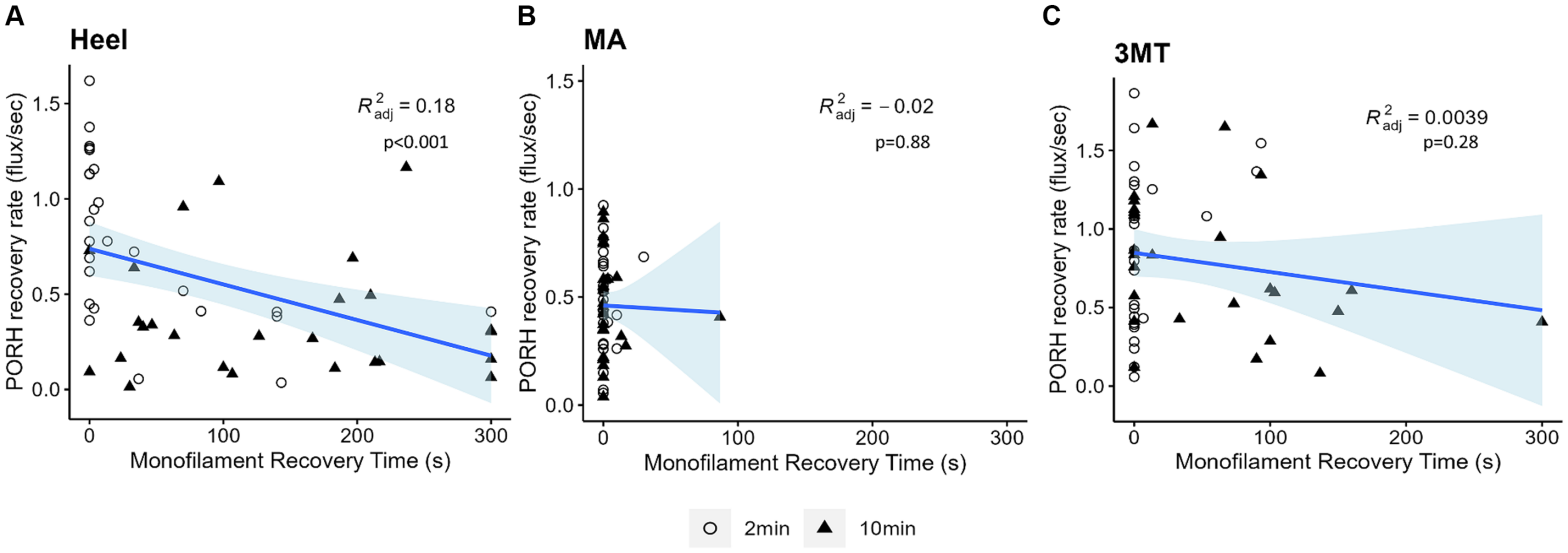
Linear regressions of PORH flux recovery rate and monofilament PT recovery time at each site **(A)** Heel **(B)** MA **(C)** 3MT. Subject’s two-minute trials are represented as open circles and ten-minute trials are represented as black triangles. Blue line and shaded area are the line of best fit and 95% confidence interval. *R*^2^ and *p*-values are reported for each site. The only significant regression was observed at the heel (*p* < 0.001) with 18% predictability.

The PT recovery time did not significantly predict peakPORH (*R*^2^ = 0.003, *F*_1,154_ = 1.435, *p* = 0.233); however, it did significantly predict PORH recovery rate (*R*^2^ = 0.127, *F*_1,154_ = 15.98, *p* < 0.001). Because both the PORH recovery rate and PT recovery time had site specific responses, regressions were fitted for each subset of data (Figure 6). Neither the MA (*R*^2^ = 0.019, *F*_1,50_ = 0.02299, *p* = 0.88) or the 3MT (*R*^2^ = 0.00393, *F*_1,50_ = 1.2, *p* = 0.278) generated any significant regression between the blood flux and skin sensitivity measures. However, the heel resulted in a statistically significant regression (*R*^2^ = 0.184, *F*_1,50_ = 12.5, *p* < 0.001), such that longer monofilament PT recovery time was found to relate to slower PORH recovery rates (*β* = −0.0019, *p* < 0.001).

## Discussion

The current work aimed to investigate the influence of a cutaneous ischemia on skin sensitivity and how it relates to microvascular reactivity. We demonstrated that localized skin ischemia on the foot sole of a healthy individual generates significant changes to skin sensitivity. As hypothesized, this change in skin sensitivity was localized to high pressure foot regions (Cleland et al., 2023) and coincided with locations where blood flow was drastically reduced during loading (3MT and heel). The sensitivity of the medial arch, which experiences very little plantar pressure during stance-like loading, did not generate a PORH response, and did not experience any significant changes to skin sensitivity. The heel had the most pronounced changes to both PORH recovery rate and PT recovery time, both of which were influenced by the duration of the ischemia. These measures in the heel were not influenced by the magnitude of ischemia, which was against our original hypothesis. Lastly, we found that the PORH recovery rate significantly predicted the PT recovery time in the heel.

### Skin sensitivity differs across the foot sole

Site differences in perceptual sensitivity have been well documented within the literature for both vibration sensitivity and monofilaments (Kekoni et al., 1989; Kennedy and Inglis, 2002; Hennig and Sterzing, 2009; Strzalkowski et al., 2015a,b; Mildren et al., 2017). Research has suggested that these differences exist due to both receptor density (Strzalkowski et al., 2018; Corniani and Saal, 2020) but also mechanical characteristics of the skin, such as thickness, hardness, and elasticity (Thomas et al., 2003; Strzalkowski et al., 2015a,b; Schmidt et al., 2018). Microneurographic data also supports the idea of regional differences in perception. Afferent firing thresholds in response to monofilaments are lower for receptors in the medial arch (greater sensitivity), compared to regions such as the fifth metatarsal and heel (Strzalkowski et al., 2018), indicating greater sensitivity to input in regions of softer and thinner skin. The monofilament thresholds generated in this current work is consistent with the existing literature. The heel was the least sensitive site (had the largest threshold) and the MA was the most sensitive.

### Longer duration of loading but not larger magnitude of loading decreases skin sensitivity

Sustained loading to the skin of the foot sole resulted in localized cutaneous ischemia and a transient decrease in monofilament sensitivity, indicated by an increased PT recovery time. Ten minutes of sustained loading generated longer PT recovery times than 2 min of sustained loading in the heel and 3MT. In other words, the PT threshold monofilament was imperceivable for longer when ischemia was induced over longer durations. Even at a small magnitude of 15% BW, the heel remained insensitive to the PT for upwards of 5 min in some individuals (Table 2). Previous work found that in upright stance, the blood flow in the heel is occluded with as little as 15% BW (Chang et al., 1996). However, the heel experiences ~20% body weight with bipedal quiet stance (Cleland et al., 2023). Therefore, the decline in sensitivity generated in the current work is particularly concerning because it suggests that quiet standing for prolonged periods (>10 min), even at low magnitudes of body weight (15% BW) can impair cutaneous sensitivity. Therefore, it is important to explore what mechanisms may be at play to generate a change in skin sensitivity with prolonged foot loading. Several of these mechanisms are briefly discussed below.

### Sustained loading could directly alter firing activity of peripheral afferents

The sustained load may have directly impacted the function of peripheral afferents and/or receptor endings within the plantar skin. Previous work has shown that an afferent’s ability to continue firing was inhibited after the prolonged mechanical input (Adriaensen et al., 1984; Handwerker et al., 1987; Ge and Khalsa, 2002); however, this inhibition was only tested in slowly adapting (SA) receptors, that have a continuous discharge throughout a load application (Johansson and Vallbo, 1979). In the current work, we used monofilaments to assess perceptual sensitivity. Monofilament perception is most closely coupled with afferent firing of fast adapting type I (FAI) receptors, associated with Meissner corpuscles (Strzalkowski et al., 2015a). Owing to the corpuscle’s elastic nature (Handler and Ginty, 2021), FAIs respond only to the initial indentation of a load and rapidly cease firing during any sustained forms of load (Ozeki and Sato, 1965; Iggo and Ogawa, 1977). Therefore, it is unlikely that the duration of a load, on its own, might differentially impact an FAI activation. However, the skin tissue which surrounds FAI endings is not elastic and acts as a filter to transmit external stimuli (Zheng and Mak, 1996; Grigg et al., 2004; Wu et al., 2006; Handler and Ginty, 2021). Therefore, it is possible that the influence of load duration on PT recovery was affected by other factors, such as mechanical properties of the skin tissue or cutaneous ischemia, rather than a change to the afferent ending itself.

### Viscoelastic properties of skin might play a role in PT recovery time

Previous work has demonstrated that skin sensitivity is influenced by mechanics of the soft tissue surrounding the receptor mechanoreceptor end organ themselves (Pubols and Pubols, 1983; Hudson et al., 2015; Strzalkowski et al., 2015b). Skin is anisotropic therefore has different stress-stain response to loading and unloading (Joodaki and Panzer, 2018). Being anisotropic would indicate that as a sustained force is removed from the foot, the deformation of the skin returns at a slower rate, also known as creep recovery. In fact, when a force was applied to the glabrous skin of a cat skin for 30 s, the skin had only recovered about 80% of its original displacement 100 s after the release of the load (Pubols, 1982). to date. In the current work, the foot skin was loaded for prolonged periods (2 or 10 min) and at upwards of 50% of BW to simulate skin deformation that would occur with bipedal stance. Although the current work did not measure skin deformation, we anticipate some residual skin deformation upon the load release. Previous work has shown a decreased discharge rates of SA afferent firing during a sustain load (Pubols and Pubols, 1983). However, it is unknown how creep recovery (or residual skin indentation) might impact a Meissner corpuscle’s (FAI) ability to generate an action potential, and thus a percept from the threshold monofilament during the current paradigm’s repetitive monofilament applications. More work in establishing the creep recovery rates in human glabrous skin is needed following typical loading observed in standing and gait to better understand its potential impact on afferent firing.

### Cutaneous blood flow contributes as a predictor of mechanoreceptor sensitivity

As hypothesized, sustained load applied to the foot sole generates a decrease in skin blood flow. Once an externally applied pressure exceeds the capillary pressure in the skin, blood flow will cease in that region (Reddy, 1986). Interestingly, the most critical contributing factor to ulceration risk in pressure sores is the duration of pressure rather than the magnitude (Agrawal and Chauhan, 2012). The current work directly supports this importance of load duration and highlights evidence for a relationship between blood flow and skin sensitivity in the foot sole. Specifically, longer load durations significantly decreased the rate of the PORH response back to baseline flux values such that blood flow remained elevated for a prolonged period following a 10-min load. This dose dependent response comes as no surprise. Previous work has demonstrated that a larger perturbation or stressor to the microvasculature generates a larger reactive response (Larkins and Williams, 1993; Chang et al., 1996; Mayrovitz et al., 1999; Petrofsky et al., 2009). However, the noteworthy finding from the current work’s regression analysis revealed that, at least at the heel, the PT recovery can significantly predict the PORH recovery rate with as much as 18% of the variability accounted for. These data indicate that a key contributor to the ability of cutaneous mechanoreceptors to generate a percept after a period of cutaneous ischemia is associated with the microvascular flow in that skin region. This may suggest that assessment of skin perception could be a marker of healthy microvascular reactivity. As we know, ambulating individuals habitually undergo the magnitudes and durations of natural loading on the foot sole and therefore routinely experiences tissue ischemia. Despite this routine ischemia, healthy feet have persevering sensory feedback and intact, healthy skin tissue.

### Clinical impact of current work

In clinical populations, such as diabetes and even healthy aging, there are morphological changes of the mechanoreceptor endings (García-Piqueras et al., 2019; García-Mesa et al., 2021), which likely contributes to their impaired function (Mackel, 1989; Mizobuchi et al., 2002; Mackel and Brink, 2003). Moreover, aging and diabetes generates chronic changes to microvascular density (Wang-Evers et al., 2021), and function (Petrofsky et al., 2009; Jan et al., 2013; Stirban, 2014; Barwick et al., 2016; Lanting et al., 2017; Balasubramanian et al., 2021). Even obesity alone has demonstrated altered microvascular reactivity (De Jongh et al., 2004) which suggests that microvascular dysfunction may prelude the cascade of changes that ultimately causes cutaneous mechanoreceptor impairment. Altering blood flow and metabolic exchange in the vasculature that immediately surround these mechanoreceptor endings is a possible contributor to the degradation to mechanoreceptor endings through disease progression. Here we have shown a relationship between skin sensitivity, and cutaneous blood flow. Interestingly, with only 15% BW, prolonged loading impacted cutaneous sensitivity in young, healthy adults. With populations that have existing balance deficits, the decline in skin sensitivity that is generated with prolonged stance would further deteriorate postural control. Impaired sensory input would reduce local pressure shifts, further prolonging loading and thus increase time of pressure induced ischemia, promoting ulcer development. The interplay between sensory afferents and microvasculature is not well understood, but it is likely that each can impact the function of the other. Future work should explore this relationship further in these clinical populations that commonly experience sensory loss, balance detriments and tissue ulceration to inform therapeutic interventions that may be able to detect or even mitigate the consequences of peripheral nerve damage.

### Limitations and considerations

There are a few limitations within the current work that must be acknowledged. With the current experimental design, the PT recovery time was only able to be tested once per site per loading condition in a participant. When averaged across the sample pool, the variability was quite extensive in some of the PT recovery times. Although the current work normalized the magnitude of force applied to the foot sole (% BW), the pressure experienced at each region would likely differ depending on individual foot shape and size. Depending on the foot architecture, how the force was dispersed across the different regions would change. Unfortunately, pressure insoles or individualized pressure data was not available for the current work. Moreover, the perception task was reported by some subjects as challenging because of pulsatile flow in foot sole interfering with ability to perceive the monofilament stimulation, particularly for the arguably largest load (10 min50% condition). Each subject was asked to report any difficulty of the task at the end of the testing session. Only a few (2/13) reported on the pulsatile flow. Importantly each of the thirteen subjects had similar trends of perception across the four loading conditions, where the largest load resulted in the longest return to sensation.

## Conclusion

In conclusion, the present results support our hypothesis that localized cutaneous ischemia from a sustained load on the foot sole impairs skin sensitivity and these changes are specific to regions of the foot sole that experience higher plantar pressures. Moreover, at the heel, there is a significant association between the skin sensitivity measures and the capillary blood flow response such that as vasodilation in the skin capillaries persist, insensitivity of the PT monofilament prevailed. Future research should capitalize on this relationship for clinical populations such as aging or diabetes to further enlighten the time course of the progression of age and diabetes related nerve damage.

## Data availability statement

The raw data supporting the conclusions of this article will be made available by the authors, without undue reservation.

## Ethics statement

The studies involving humans were approved by University of Guelph Research Ethics Board. The studies were conducted in accordance with the local legislation and institutional requirements. The participants provided their written informed consent to participate in this study.

## Author contributions

EH: Conceptualization, Data curation, Formal analysis, Funding acquisition, Investigation, Methodology, Resources, Software, Validation, Visualization, Writing – original draft, Writing – review & editing. MA: Data curation, Formal analysis, Writing – review & editing. LB: Conceptualization, Funding acquisition, Methodology, Resources, Supervision, Validation, Visualization, Writing – review & editing.
